# Supplementary material for: Identification and functional analysis of non-coding regulatory small RNA FenSr3 in Bacillus amyloliquefaciens LPB-18
Source: PeerJ. 2023 May 15;11:e15236. doi: 10.7717/peerj.15236 (PMC10194069; doi:10.7717/peerj.15236)
Supplement: Supplemental Information 4 [file peerj-11-15236-s004.zip › KO/CK-vs-T1_map/map04144.html]

KEGG PATHWAY: Endocytosis - Reference pathway


|  |  |
| --- | --- |
| **Endocytosis - Reference pathway** |  |

[
Pathway menu
| Organism menu
| Pathway entry
| Show description
| User data mapping
]

Endocytosis is a mechanism for cells to remove ligands, nutrients, and plasma membrane (PM) proteins, and lipids from the cell surface, bringing them into the cell interior. Transmembrane proteins entering through clathrin-dependent endocytosis (CDE) have sequences in their cytoplasmic domains that bind to the APs (adaptor-related protein complexes) and enable their rapid removal from the PM. In addition to APs and clathrin, there are numerous accessory proteins including dynamin. Depending on the various proteins that enter the endosome membrane, these cargoes are sorted to distinct destinations. Some cargoes, such as nutrient receptors, are recycled back to the PM. Ubiquitylated membrane proteins, such as activated growth-factor receptors, are sorted into intraluminal vesicles and eventually end up in the lysosome lumen via multivesicular endosomes (MVEs). There are distinct mechanisms of clathrin-independent endocytosis (CIE) depending upon the cargo and the cell type.

|  |  |  |
| --- | --- | --- |
| Reference pathway | 184% 150% 122% 100% 82% 67% 55% | 图片下载 |
